# Pericentromeric Non-Coding DNA Transcription Is Associated with Niche Impairment in Patients with Ineffective or Partially Effective Multiple Myeloma Treatment

**DOI:** 10.3390/ijms23063359

**Published:** 2022-03-20

**Authors:** Natella I. Enukashvily, Natalia Semenova, Anna V. Chubar, Dmitry I. Ostromyshenskii, Ekaterina A. Gushcha, Sergei Gritsaev, Stanislav S. Bessmeltsev, Viktor I. Rugal, Egor M. Prikhodko, Ivan Kostroma, Anastasia Zherniakova, Anastasia V. Kotova, Liubov A. Belik, Alexander Shumeev, Irina I. Maslennikova, Dmitry I. Ivolgin

**Affiliations:** 1 Lab of the Non-Coding DNA Studies, Institute of Cytology, Russian Academy of Sciences, 194064 St. Petersburg, Russia; annachubar95@incras.ru (A.V.C.); necroforus@gmail.com (D.I.O.); gushcha.ekaterina@gmail.com (E.A.G.); anastkotova@gmail.com (A.V.K.); l.a.belik@list.ru (L.A.B.); 2 Cell Technologies Lab, North-Western State Medical University Named after I.I. Mechnikov, 191015 St. Petersburg, Russia; Irina.Maslennikova@szgmu.ru (I.I.M.); Dmitrii.Ivolgin@szgmu.ru (D.I.I.); 3 Clinical Department, Russian Research Institute of Hematology and Transfusiology FMBA of Russia, 191024 St. Petersburg, Russia; gritsaevsv@mail.ru (S.G.); bessmeltsev@yandex.ru (S.S.B.); sciencerugal@gmail.com (V.I.R.); obex@rambler.ru (I.K.); nasta2045@yandex.ru (A.Z.); 4 Pokrovsky Stem Cell Bank, LLC, 199106 St. Petersburg, Russia; ceo@pokrovcell.ru (E.M.P.); a-shu@yandex.ru (A.S.); 5 Faculty of Clinical Propaedeutics, North-Western State Medical University Named after I.I. Mechnikov, 191015 St. Petersburg, Russia

**Keywords:** multiple myeloma, mesenchymal stromal cells, microvessels, tumor microenvironment, long non-coding RNA, human satellite 3, human satellite 2, non-coding DNA transcripts

## Abstract

Mesenchymal stromal cells (MSC) ‘educated’ by tumor cells are an essential component of the multiple myeloma (MM) tumor microenvironment (TME) involved in tumor progression. Transcription of tandemly repeated (TR) non-coding DNA is often activated in many tumors and is required for tumor progression and cancer cells genome reorganization. The aim of the work was to study functional properties including the TR DNA transcription profile of MSC from the hematopoietic niche of treated MM patients. Healthy donors (HD) and patients after bortezomib-based treatment (with partial or complete response, PoCR, and non-responders, NR) were enrolled in the study. Their trephine biopsies were examined histologically to evaluate the hematopoietic niche. MSC cultures obtained from the biopsies were used for evaluation of the proliferation rate, osteogenic differentiation, presence of tumor MSC markers, resistance to bortezomib, and pericentromeric TR DNA transcription level. The MSC ‘education’ by multiple myeloma cells was mimicked in co-culture experiments with or without bortezomib. The TR DNA transcription profile was accessed. The histological examination revealed the persistence of the tumor microenvironment (especially of the vasculature) in treated patients. In co-culture experiments, MSC of bortezomib-treated patients were more resistant to bortezomib and protected cancer MM cells of the RPMI8226 cell line more effectively than HD-MSC did. The MSC obtained from PoCR and NR samples differed in their functional properties (proliferation capacity, osteogenic potential, and cancer-associated fibroblasts markers). Transcriptome analysis revealed activation of the TR transcription in cells of non-hematopoietic origin from NR patients’ bone marrow. The pericentromeric TR DNA of HS2/HS3 families was among the most upregulated in stromal MSC but not in cancer cells. The highest level of transcription was observed in NR-MSC. Transcription of HS2/HS3 was not detected in healthy donors MSC unless they were co-cultured with MM cancer cells and acquired cancer-associated phenotype. Treatment with TNFα downregulated HS2/HS3 transcription in MSC and upregulated in MM cells. Our results suggest that the hematopoietic niche retains the cancer-associated phenotype after treatment. Pericentromeric non-coding DNA transcription is associated with the MSC cancer-associated phenotype in patients with ineffective or partially effective multiple myeloma treatment.

## 1. Introduction

Multiple myeloma (MM) is a malignant hematopoietic disease characterized by uncontrolled plasma cell (PC) expansion in the bone marrow (BM) and by the presence of monoclonal immunoglobulin in the blood and/or urine [1,2,3]. MM accounts for 13% of all hematologic cancers and remains incurable despite new treatment approaches and drugs development [2,4]. MM growth and development strongly depend not only on the large number of genetic instabilities identified in tumor cells [5] but also on the interaction between cancer cells and tumor microenvironment (TME) that consists of various cell types such as osteoblasts, osteoclasts, adipocytes, endothelium, immune cells and mesenchymal stromal cells (MSC). MSC of normal and cancer hematopoietic niches play a crucial role in regulating the proliferation and differentiation of hematopoietic stem cells by producing a number of cytokines and growth factors [3,6,7]. MM cells alter TME providing favorable conditions for the tumor to grow and to avoid immune surveillance [1,8,9,10,11,12]. MSC ‘educated’ by tumor cells are one of the key elements of TME. There is evidence of the MSC role in the chemoresistance emergence. TME MSC are resistant to anticancer therapy and contribute to early relapse and survival of residual cancer cells. The mechanism of MSC transition to TME MSC induced by tumor cells is one of the key questions in the study of the early recurrences and development of resistance to the chosen methods of therapy. However, the hematopoietic niche after treatment is underinvestigated. It is not fully understood whether the niche returns to its normal state or some changes still persist.

Though the mechanism of MM progression and TME induction and organization by cancer cells are not fully understood, there is a body of evidence suggesting the role of long non-coding RNAs (lncRNA) in the onset of the disease [13]. The human genome project revealed that only about 1–5% of DNA is made up of protein-coding genes; the other 95–99% is non-coding. However, most of the non-coding DNA is transcriptionally active [14]. Non-coding RNA molecules may be divided into two groups: short non-coding (<200 bp) and long non-coding RNAs (>200 bp, lncRNAs) [15]. Most of the lncRNA are functionally relevant; they are involved in the regulation of all the processes in a living cell. The lncRNA transcription from the tandemly repeated (TR) DNA of pericentromeric human satellite 2 (HS2) and 3 (HS3), which are closely related to each other, is shown to increase by 10 times in solid tumors [16]. The TR satellite DNA is a part of constitutive heterochromatin and resides in the centromere and pericentromere (periCEN) regions of chromosomes. Transcripts of pericentromeric TR DNA play an important role in tumor progression. They are associated with poor prognosis [16,17]. Transcription of repetitive DNA in solid tumors is accompanied by hypomethylation [18,19]. The hypomethylation of satellite DNA and other repetitive elements has also been demonstrated in MM primary cell cultures [20]. However, the transcriptional status of non-coding TR DNAs such as HS2 and HS3 (HS2/HS3) has not been examined in MM and other hematological malignancies.

The aim of our study was to explore functional properties including the TR DNA transcription profile of MSC from the hematopoietic niche of treated MM patients with different responses to treatment.

In the present study, we demonstrated that the TME of treated patients retained its changed characteristics and did not return to a normal state of its microenvironment. MSC of TME protected cancer plasma cells from bortezomib increasing their viability. TR DNA transcription was upregulated in MSC of TME. Pericentromeric satellite DNA of HS3 was the most upregulated TR in MSC of TME and its transcription can be induced in healthy donors’ MSC by co-culturing with MM cells. The transcription was downregulated by pro-inflammatory cytokines, TNFα and IL-6. The phenotype of the MSC from MM patients with partial or complete response (PoCR) to treatment (minimal residue disease (MRD) < 5%) differed from the phenotype healthy donors’ (HD) MSC being closer to the cancer-associated fibroblasts’ (CAF) phenotype. The MSC of patients that did not respond to the immunotherapy, chemotherapy, and autologous hematopoietic stem cell transplantation (autoHCT) had a prominent cancer-associated phenotype. The upregulation of pericentromeric DNA transcription in cancer-associated MM-MSC of non-responders (NR) observed in our study was not demonstrated previously.

## 2. Results

### 2.1. Hematopoietic Niche Retains Cancer-Associated Attributes after Treatment

#### 2.1.1. Histological Analysis of MM TME

**Figure 1:**
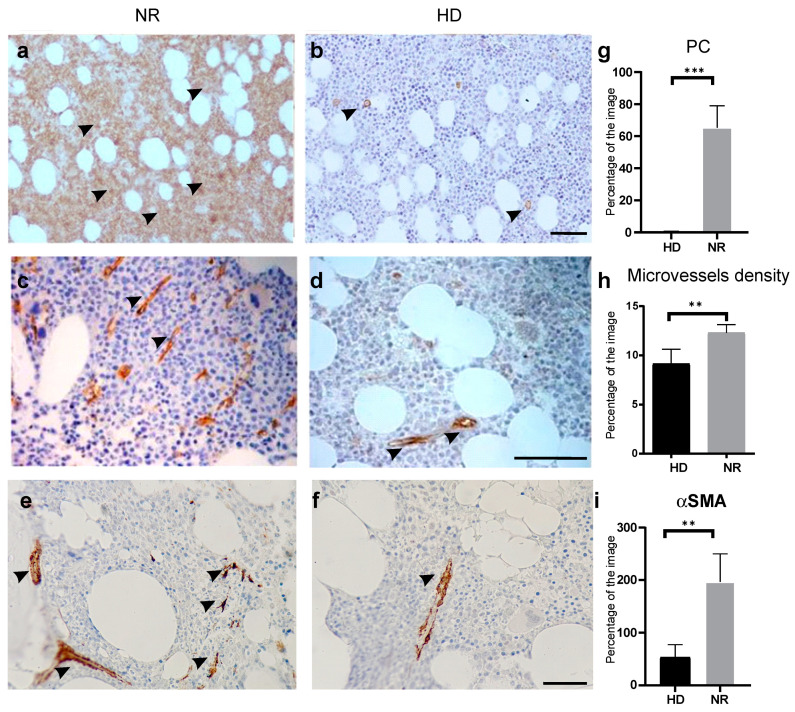
Histopathological examination of BM from HD and NR patients. (**a**,**b**) IHC detection of PC (brown color) in BM of NR patient (**a**) and HD (**b**) with an antibody against CD138. (**c**,**d**) IHC detection of microvessels (brown color) in BM of NR patient (**c**) and HD (**d**) with an antibody against CD34. (**e**,**f**) IHC detection of α-SMA+ cell (brown color) in BM of NR patient (**e**) and HD (**f**). The scale bar–100 µm. (**g**–**i**) Quantifications of corresponding IHC. The percentage of the images’ area covered by the IHC staining is plotted on Y-axis. The data are shown as mean ± SD. ** *p* < 0.01, *** *p* < 0.001. Abbreviations: BM = bone marrow; HD = healthy donors; NR = non-responder; PC = plasma cells, IHC = immunohistochemical staining. Arrows are pointing the areas of positive staining.

The morphological features of the BM microenvironment were studied by histological methods, including endosteal and endothelial hematological niches in patients with MM (Figure 1). After morphological examination, key morphological elements were identified that indicated a change in the state of a hematological niche: the number of active cells on the endost, the density of microcirculation vessels, the expression of the CXCR4 protein (a prognostic marker for MM), the detection of cells, positively stained with an antibody against alpha smooth muscle actin (αSMA+, a marker of TME MSC and cancer-associated fibroblasts, CAF).

Increased microvascular density in BM stromal TME was revealed in all the PoCR and NR patients. An increased number of microcirculation vessels was found in 10 of 12 patients, including all patients from the group of NR patients. The microvessels density was measured as 9.14 ± 1.47% of field of view for HD patients and 12.30 ± 0.82% for the NR patients by histological software (Table 1, Figure 1c,d,h). The reference value for the HD, in the laboratory practice, is 7.1 ± 4.1% according to the long-term statistic observations. The microvessels density in BM of both NR and PoCR patients differed significantly from the reference values (*p* < 0.05).

Increased angiogenesis correlated with the number of PC in the myelogram (r = 0.58; *p* < 0.05) and with the type of BM infiltration (r = 0.85; *p* < 0.05), as well as with osteodestructive changes in the patients’ clinical records (r = 0.65; *p* < 0.05).

As a result of the morphological examination, differences in the number of cells on the endost were also revealed in patients of the various groups. In the group of PoCR patients, the number of cells in the endosteal zone was significantly higher (2.7 ± 0.3 cells per unit length of the trabecula) than in NR patients (1.9 ± 0.2 cells per unit length of the trabecula) (*p* = 0.038, Student’s *t*-criterion). During histological examinations, there were places revealed that contained active osteoblasts and active stromal cells with an increased number of microvessels observed next to them.

αSMA+ cells were observed in all studied samples as part of BM microvessels. These cells represented αSMA+pericytes. In addition, in all the NR groups and in one PoCR patient, single cells lying in the thickness in the hematopoietic tissue (αSMA+ MM-MSC) were determined (Figure 1e,f,i).

The expression of the CXCR4 protein showed no significant differences between the samples.

Thus, morphological studies revealed key changes in the histology of the hematological niche in patients with MM.

#### 2.1.2. MSC and RPMI8226 Viability in the Presence of Bortezomib

**Figure 2:**
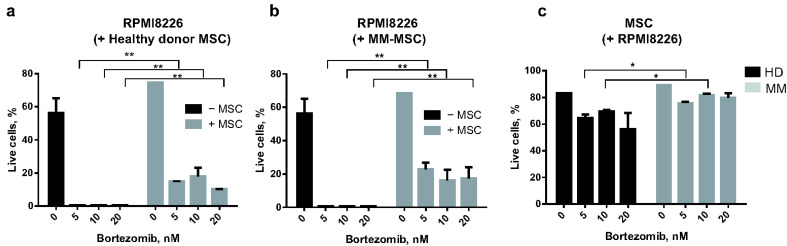
MSC from HD (**a**) or MM patients (**b**) supported the survival of MM cells of RPMI8226 cell line during treatment with bortezomib, a standard medication to treat MM. RPMI8226 were grown in non-contact co-culture with HD-MSC or MM-MSC in the presence of bortezomib for 48 h. Then, RPMI8226 (**a**,**b**) and MSC (**c**) were harvested, stained with annexin-FITC and propidium iodide and subjected to flow cytometry to reveal live and apoptotic cells. *X*-axis—bortezomib concentration, nM; *Y*-axis—the percentage of live cells defined as cells negatively stained with both annexin and propidium iodide. ** *p* < 0.01, * *p* < 0.05. Abbreviations: MSC = mesenchymal stem cells; HD-MSC = mesenchymal stem cells of healthy donors; HD = healthy donors; MM = multiple myeloma; RPMI8226 = a MM cell line (RPMI8226).

MSC are one of the main components of the hematopoietic niche in health and disease. Tumor-educated MSC protect cancer cells from cytostatic drugs as shown in many types of tumors but not in MM. We addressed the question, of whether MSC from primary cultures helped cancer cells to evade drug-induced apoptosis. The interaction between MSC and MM cancer cells was mimicked in non-contact co-culture experiments when MM cell line RPMI8226 was co-cultured with primary MSC cultures of MM patients and HD. Bortezomib, a first-in-class proteasome inhibitor, approved for the frontline treatment of newly diagnosed MM, was used in a range of concentrations from 5 to 20 nM. According to the published data, RPMI8226 is highly sensitive to the drug, with an IC50 between 1.9 and 10.2 nM, when incubated for 48 h [21]. Therefore, a range of concentrations from 5 to 20 nm was chosen for a 48 h incubation. In this range, bortezomib-induced apoptosis in more than 99% of RPMI8226 cells when cells were grown without MSC. However, the number of live cells increased significantly when MM cells were grown in the presence of MSC obtained either from HD or MM patients (Figure 2a,b). When bortezomib was added in a low concentration (5 nm), the protective effect of MSC obtained from MM patients was more prominent as compared to HD-MSC (RPMI8226 live cells percentage: 22.9 ± 3.9 vs. 14.9 ± 0.2%, *p* < 0.05) (Figure 2c). MSC themselves were resistant to bortezomib (Figure 2c). MM-MSC were more resistant to 5–20 nM bortezomib than HD-MSC were (Figure 2c).

Thus, MSC, especially those obtained from MM patients, are more resistant to bortezomib than cancer MM cells. Their presence increases the survival rate of MM cells. The MSC from MM hematopoietic niche are more effective in the protection of cancer cells from an anti-MM drug, bortezomib as compared to MSC from the normal hematopoietic niche of HD. According to our data, MSC of the tumor hematopoietic niche can be involved in the drug resistance of MM cells. The bortezomib treatment does not eliminate the MSC component of the niche.

In the next step, we accessed the differences between MSC from the hematopoietic niche of healthy donors and treated MM patients.

#### 2.1.3. HD and MM-MSC: Comparison of Functional Properties

During the histological examination, the BM hematopoietic niche in all the samples obtained from treated patients was different from the BM niche of the HD despite the treatment and its outcome (Figure 1). Hematopoietic niche is a multicomponent structure but MSC are considered as its key player. TME MSC are involved in drug resistance and they survive the treatment (Figure 2). In the next step, we addressed the question of whether MSC of HD and different groups of treated patients differ in their functional properties such as their proliferation capacity, a set of surface markers, and differentiation capabilities. Therefore, MSC from these samples were cultured and their functional properties were accessed.

- Surface markers of RPMI8226 and MSC.

The cell line RPMI8226 deposited in ATCC collection was obtained from a patient with confirmed MM. However, it has been established as a cell line for a long time. Therefore, the expression of MM surface markers was evaluated to check whether the cell line can be used in co-culture experiments as MM cells. The cell line RPMI8226 used in our study contained >50% of CD38+CD138+ cells (i.e., PC). However, all cells were positive for CD138 (a marker of PC and many neoplasia). Immunophenotype of all cells corresponded to the immunophenotype of abnormal PC: (CD56high, low or no expression for CD45, CD19, CD27, and CD81).

Most of the MSC cultures met the ISCT criteria with more than 95% of the cells being positive for MSC markers in cell cultures and less than 2% being positive for negative MSC markers (Table S1).

No significant difference between MM-MSC and HD-MSC was found. In the group of MM-MSC from PoCR patients, the number of macrophages was slightly increased to 3.3 ± 3.2% in comparison with 1.5 ± 0.8% of HD-MSC, but these cells disappeared after passaging as long as they could not divide in culture.

Thus, the MSC of all samples retained the set of basic MSC surface markers.

- The proliferation rate of MM-MSC

**Figure 3:**
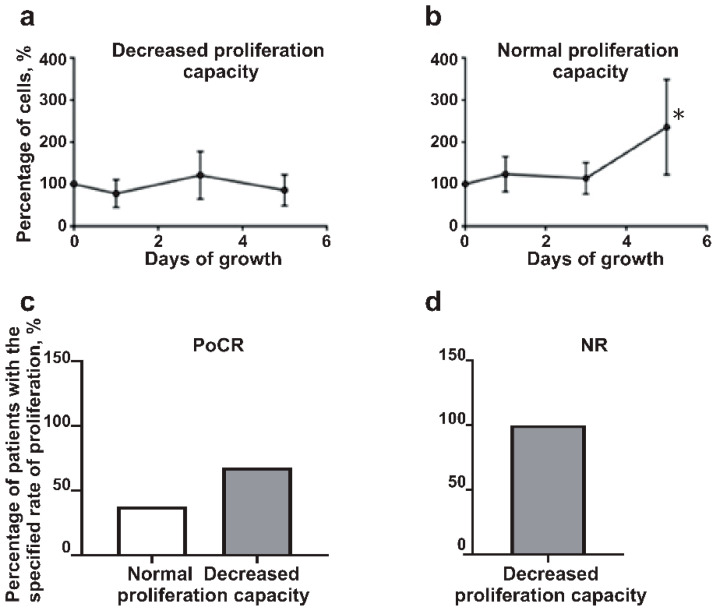
The proliferation rate of MM-MSC and HD-MSC. MSC cultures from patients were divided into groups with decreased (**a**) and normal proliferation capacity (**b**); (**c**) part of PoCR patients’ MSC cultures had normal proliferation rate (white column) while the majority of samples had decreased proliferation capacity (grey column); (**d**) all of NR proliferated at a slower rate (similar to the rate shown in (**a**). Abbreviations: NR = non-responders, patients with ineffective treatment; PoCR = partial or complete response, MSC = mesenchymal stem cells, HD-MSC = mesenchymal stem cells of healthy donors; MM-MSC = mesenchymal stem cells from bone marrow of patients with multiple myeloma. * *p* < 0.05.

MM-MSC cell cultures were expanded during 4–5 passages in vitro. Then the cell proliferation decreased and the cells acquired a senescence-associated phenotype. In our study, the expanded MSC from BM samples could be divided into two groups: the samples with the rate of proliferation similar to HD-MSC (‘normal rate’) and those that exhibited a much lower proliferative capacity (Figure 3a,b).

The group of MM-MSC with the normal rate of proliferation included 37.5% of PoCR patients. All MSC cultures from the NR and 62.5% of PoCR patients proliferated at a slower rate (Figure 3c,d). Two-thirds of these slowly proliferating PoCR MSC samples were obtained from patients who had positive MRD status. Hence, the MSC from NR patients and even from the majority of successfully treated patients differed from HD-MSC in their proliferation capacity.

Thus, the proliferation rate of MSC from the hematopoietic niche of treated patients was decreased.

- The osteogenic differentiation of MSC from PoCR and NR patients

**Figure 4:**
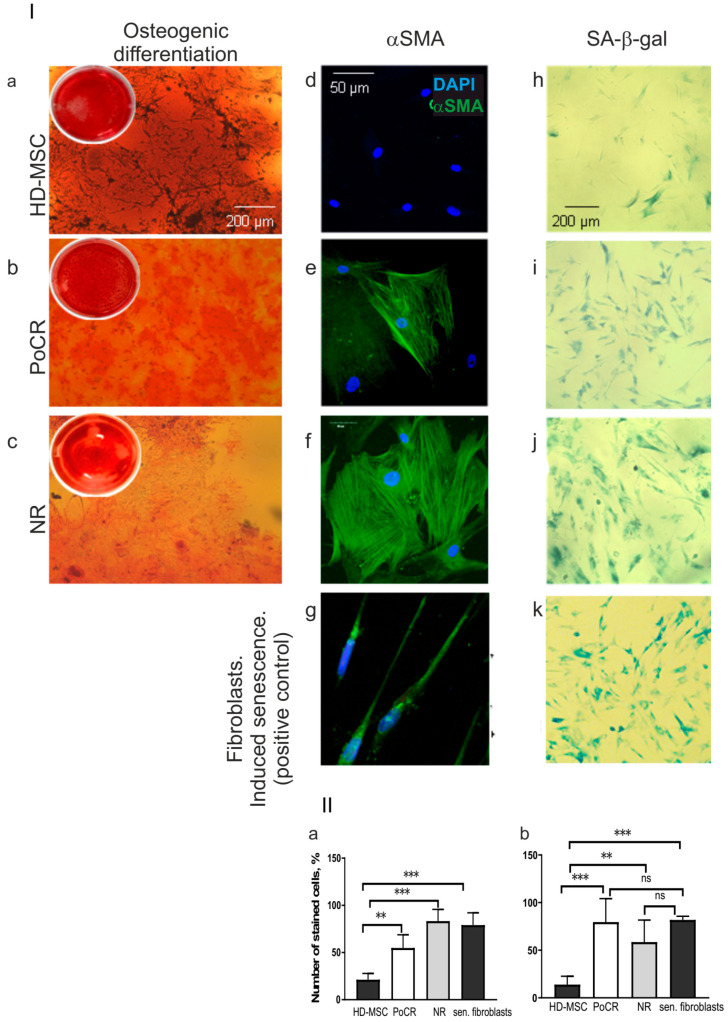
Osteogenic potential (**a**–**c**), TME markers (**d**–**k**), in HD-MSC (**a**,**d**,**h**), PoCR MSC (**b**,**e**,**i**), and NR-MSC (**c**,**f**,**j**). Fibroblasts with the drug-induced senescence were used as a positive control (**g**,**k**). Panel I: (**a**,**b**,**c**) Calcifications revealed by staining with Alizarin Red, insets in upper left corners of each image represent a general view of a plate well; (**d**–**g**) staining with an antibody against αSMA (green), nuclei are counterstained with DAPI; (**h**–**k**) activated SA-β-gal staining; nuclei are counterstained with DAPI. The scale bars for each panel are shown in the images. Panel II: The results of staining quantification: number of cells stained with (**a**) an anti αSMA antibody or (**b**) with a dye revealing activated SA-β-gal is plotted on the Y-axis. ** *p* < 0.01, *** *p* < 0.001, ns = non-significant (*p* > 0.05). Abbreviations: DAPI = 4’,6-diamidino-2-phenylindole; HD-MSC = mesenchymal stromal cells from healthy donors; HS2/HS3 = human satellite 2,3; MSC = mesenchymal stromal cells; NR = non-responder; PoCR = partial or complete response; SA-β-gal = senescence-associated β-galactosidase; TME = tumor microenvironment; αSMA = α-smooth muscle actin.

Bone formation impairment resulting in osteolytic lesions is one of the distinct features of the MM. Bone disease is caused by defective osteogenic differentiation of MM-MSC [2,3]. Therefore, we decided to test the osteogenic potential of obtained MSC cultures to check whether it is restored after treatment (Figure 4).

Calcifications occupied up to 80–90% of the field of view in the group of osteogenically induced HD-MSC (Figure 4, Panel I, a). MM-MSC from PoCR patients maintained their capability to osteogenic differentiation but displayed diversity between patients (Figure 4, Panel I, b). MM-MSC from NR had reduced capability to differentiate into osteogenic cells (Figure 4, Panel I, c).

- Cancer-associated phenotype of MM-MSC

The decreased proliferation rate that we observed in MM-MSC samples is typical for TME MSC [22]. Thus, MSC were further checked for such TME MSC markers as αSMA and SA-β-gal.

Alpha smooth muscle actin (αSMA) can be revealed in MM BM and appears in MM-MSC in vitro after treatment with MM-derived exosomes and acquires some features of CAF-associated fibroblasts [23,24,25]. In HD-MSC, αSMA protein was revealed in less than 15% of the cells and did not form well-shaped actin fibers (Figure 4, panel I, d). However, αSMA fibrils were detected in 100% of cell cultures from NR and all those cultures from the PoCR patients that had a decreased proliferation rate (Figure 4a,e,f panel I,II).

Activated SA-β-gal is a marker of MM-MSC that acquires a senescent phenotype to increase their tumor-supportive capacities [1,26]. It was detected in 14.0 ± 4.6% of HD-MSC. In cell cultures of both MM groups (PoCR and NR), the number of SA-β-gal positive cells was significantly higher—75.8 ± 10.4% and 64.9 ± 12.4, respectively. In fibroblasts treated with doxorubicin at the senescence-inducing concentration (0.1 μg/mL), 81.2 ± 1.8% of cells were positively stained for SA-β-gal (Figure 4b,h–k, panel I,II).

According to the data obtained, the hematopoietic niche does not return to its normal state. We demonstrated this at both the tissue and cellular level.

### 2.2. Transcription of Tandemly Repeated DNA Is Increased in MM-MSC

**Figure 5:**
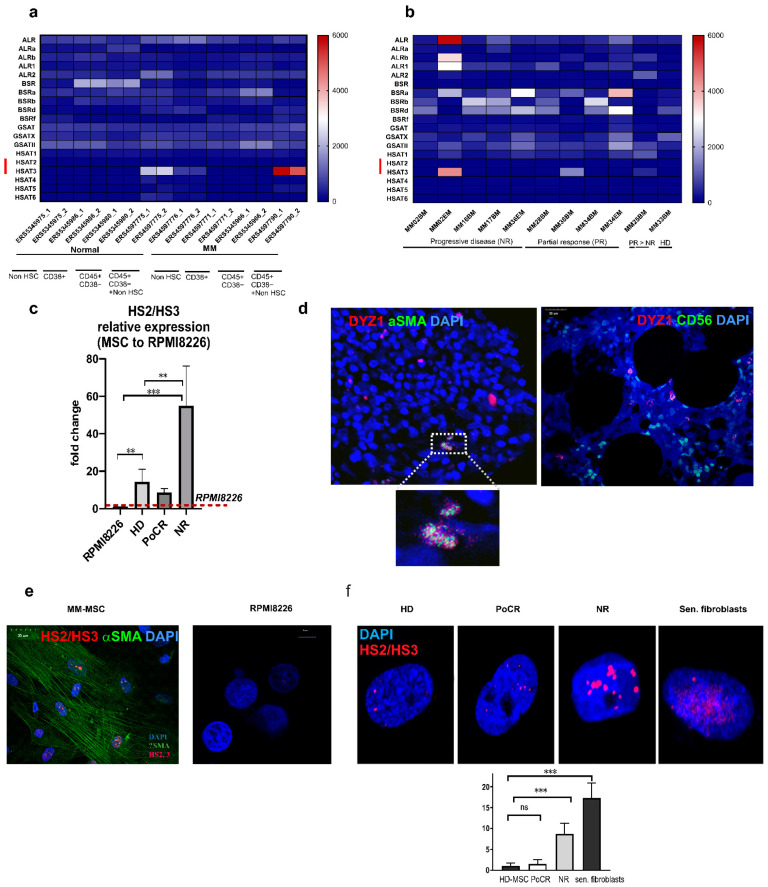
HS2/HS3 TR DNA transcription in MM-MSC. (**a**) A heatmap of TR transcripts TPM (transcripts per million) in single-cell transcriptomes of different BM populations; (**b**) a heatmap of TR transcripts TPM in single-cell MM transcriptomes of patients with different disease; TR DNA families abbreviations: ALR = α-satellite repeats, BSR = β-satellite repeats, GSR = γ-satellite repeats, HSR = human satellite repeats; red lines mark HS2/HS3 families. (**c**) qPCR of cDNA samples from NR-MSC, PoCR MSC, HD-MSC, and RPMI8226; fold change is plotted on the Y-axis, the red dashed line marks the value of transcription in RPMI8226 set as 1; GAPDH was used as a reference gene. (**d**) Pericentromeric HS2/HS3 transcripts revealed in BM trephines of MM patients by DNA–RNA FISH (red). Samples were co-stained (green) either with an anti αSMA (to reveal αSMA+ MSC) or anti-CD56 antibody (to reveal cancer cells). (**e**) After trephines examination, HS2/HS3 transcripts (red) were probed in MSC and RPMI8226 grown in vitro by DNA–RNA FISH; MSC were co-stained with an antibody against αSMA (green). (**f**) HS2/HS3 transcription in MM-MSC of patients with different responses to treatment. The results of quantification are given below the images. Nuclei in (**d**–**f**) were counterstained with DAPI. The scale bar is indicated in the images. Abbreviations: DAPI = 4’,6-diamidino-2-phenylindole; FISH = fluorescence in situ hybridization; HS2/HS3 = human satellite 2,3; MSC = mesenchymal stromal cells; PoCR = partial or complete response; αSMA = α-smooth muscle actin. ** *p* < 0.01, *** *p* < 0.001, ns = non-significant (*p* > 0.05).

We demonstrated that MM-MSC of treated patients differed from HD-MSC. Then we aimed to investigate, whether the transcription profile of non-coding TR DNA of pericentromeric regions is changed in hematopoietic malignancies in the same way as was shown in solid tumors [16,18,27] where it can be attributed either to cancer cells [16,18,27] or to cancer-associated fibroblasts as we demonstrated in pilot experiments [28]. Recently, single-cell transcriptomes of BM samples obtained from the different groups of MM patients were published by Ryu et al. (2020) [29] and by de Jong et al., (2021) [11]. We accessed the TR transcription in these transcriptomes (Figure 5a,b). MM transcriptomes published by de Jong et al. (2021) [11], were enriched in pericentromeric tandem repeats transcripts of the HS3 family, especially in the pooled fraction of CD45+/CD38− cells and cells of non-hematopoietic origin (Figure 5a, ERS459790_1 and_2) and in the fraction of cells of non-hematopoietic origin that included endothelial cells and mesenchymal stromal cells gated by CD271, CD105, CD31, and CD34 (Figure 5a, ERS4597776_1 and 2). Transcription of HS3 DNA, as well as other TR, was more prominent in patients, who had not responded to treatment (Figure 5b).

According to the transcriptomes’ analysis, CD38+ MM cells were not the main producers of TR transcripts in MM samples (Figure 5a, CD38+ transcriptomes). The qPCR results (Figure 5c) confirmed the result obtained in silico. The level of HS2/HS3 transcription in RPMI8226 was the lowest compared to all the MM-MSC groups. Fold change values in all MSC groups (HD-MSC: 14.68 ± 6.8; PoCR: 8.6 ± 2.1; NR: 54.9 ± 21.2) were calculated with RPMI8226 HS2/HS3 transcription level set as 1 (Figure 5c). The level of HS2/HS3 transcription in NR patients MSC was the highest demonstrating the same trend as observed in BM transcriptomes in silico (Figure 5b).

At the next step, the localization of HS2/HS3 transcripts was accessed both in trephines and in cultured MM-MSC and RPMI8226 by DNA–RNA FISH (Figure 5d,e). Human satellites are chromosome specific. Therefore, before the DNA–RNA FISH, the chromosome specificity of the HS2/HS3 probe (DYZ1) was studied on metaphase spreads with DNA-FISH. An oligonucleotide probe we used hybridized to most of the pericentromeric regions in conditions described in the Materials and Methods section, as shown by DNA–DNA FISH on metaphase spreads (Figure S1). Therefore, we used it in subsequent DNA–RNA FISH studies to reveal HS2/HS3 transcripts in cells. In trephines, CD56+ cells did not contain hybridization signals, while αSMA positive cells (MSC of TME) were enriched in them. (Figure 5d). The result was confirmed in cells grown in culture. MM-MSC from treated patients were positively stained for αSMA confirming that they belong to the TME MSC pool that survived the treatment. Most of the cells stained with the antibody also contained HS2/HS3 transcripts as revealed by DNA–RNA FISH (Figure 5e). However, RPMI8226 cells, originating from MM plasmatic cells, were free of the hybridization signal (Figure 5e).

Thus, we concluded that the HS2/HS3 burst of transcription occurred not in MM cells but rather in MM-MSC cells and is a part of the cancer-associated MSC phenotype.

### 2.3. Pericentromeric Satellite DNA Transcription in MM-MSC of Patients with Different Response to Treatment

Pericentromeric TR DNA is transcriptionally active in many tumors [16,30,31,32]. According to our previously obtained data, cancer-associated fibroblasts and MSC are the main contributors to this transcriptional burst in some solid tumors. In hematological malignancies, the level of transcription was not evaluated. We demonstrated in silico that TR transcription, including HS families, was elevated in the BM of patients who did not respond to treatment (Figure 5b) and to a lesser extent, in patients with partial response. In situ, we observed HS2/HS3 transcripts in MM-MSC using combined DNA–RNA FISH and immunostaining (Figure 5d,e). Further study revealed that the level of transcription in MM-MSC grown in vitro followed the same pattern as we observed in silico for the whole MM BM transcriptomes with the maximal transcription in NR-MSC and the lowest level of HS2/HS3 transcription in HD-MSC (Figure 5f).

### 2.4. HS2/HS3 Transcription Is Induced in MM-MSC during Co-Cultivation with RPMI 8226

**Figure 6:**
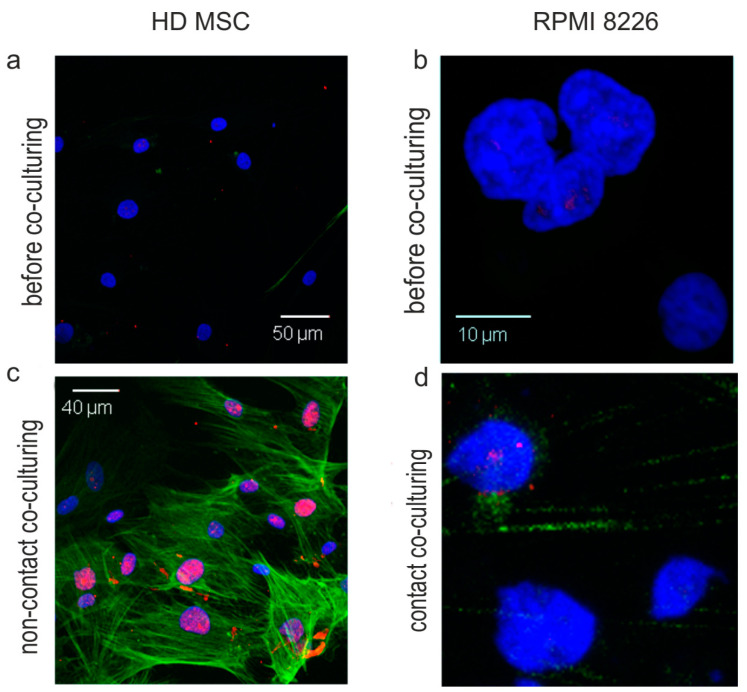
HS2/HS3 transcription (red) and αSMA expression (green) in HD-MSC (**a**,**c**) and RPMI (**b**,**d**) before (**a**,**b**) and after (**c**,**d**) non-contact (**c**) and contact (**d**) co-culturing. In contact conditions, RPMI8226 cells tended to attach to MSC. Therefore, in (**d**) RPMI 8226 cells are shown with a background of MSC αSMA fibers. The scale bars are shown on the images. Abbreviations: DAPI = 4ʹ,6-diamidino-2-phenylindole; HD-MSC = mesenchymal stromal cells from healthy donors; HS2/HS3 = human satellite 2/3; MSC = mesenchymal stromal cells; αSMA = α-smooth muscle actin.

HS2/HS3 transcription occurred in MM-MSC, especially in NR-MSC (Figure 5b,f), and was associated with the phenotype of TME MSC. We studied, whether the transcription could be induced in MSC by interaction with MM cells that did not transcribe HS2/HS3 themselves. The process of MSC ‘education’ by MM cells was mimicked in co-culture experiments. HD-MSC were co-cultured with RPMI8226 cells. Then MSC were stained with the anti αSMA antibody to prove the TME MSC phenotype and hybridized with the HS2/HS3 DYZ1 probe (Figure 6a,c). Both immunostaining and hybridization signals were absent in HD-MSC before co-culturing. However, αSMA regular well-shaped fibers and HS2/HS3 transcripts were revealed in HD-MSC after non-contact co-culturing with RPMI8226 (Figure 6c) but not in RPMI8226 cells themselves even after contact co-culturing (Figure 6d).

Thus, MM cells can induce a cancer-associated phenotype in HD-MSC even in the absence of cell-to-cell contact and this phenotype includes the increased level of pericentromeric non-coding TR DNA transcription.

### 2.5. HS2/HS3 Transcription Is Downregulated in MM-MSC but Upregulated in RPMI8226 after Treatment with TNF-a

HS2/HS3 transcription occurred in MM-MSC, especially in NR-MSC (Figure 5b,f), and was associated with TME MSC phenotype (Figure 5 and Figure 6). Cytokines IL-6 and VEGF are involved in TME MSC formation and in MM progression as key players [1,33]. Recently, Tom Cupedo’s group identified inflammatory MSC (iMSC) specific to MM bone marrow and involved in immune cell-mediated stromal inflammation [11]. Development of this subtype of TME MSC was the result of activation caused by inflammatory mediators activating NF-κB signaling, such as tumor necrosis factor (TNF).

**Figure 7:**
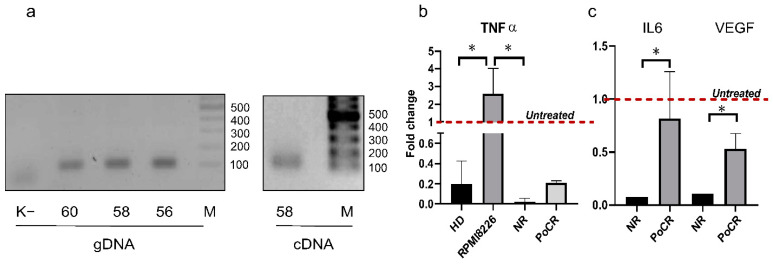
Influence of proinflammation cytokines (TNFα and IL-6) and VEGF on HS2/HS3 transcription. (**a**) Primers verification by PCR on genomic DNA (gDNA) and cDNA. MW marker is shown on the right sides of the gels, temperatures of annealing are shown below the images; (**b**) qPCR of HS2/HS3 transcripts in cells treated with TNFα, in each group transcription in untreated cells was set as 1 (red dashed line); (**c**) qPCR of HS2/HS3 transcripts in cells treated with IL-6 or VEGF, in each group transcription in untreated cells was set as 1 (red dashed line). * *p* < 0.05.

Taking into account the important role of these cytokines in TME formation, we accessed their influence on HS2/HS3 transcription in MM-MSC and RPMI8226 by qPCR. HS sequences are tandemly repeated, therefore many HS primers amplify several sequences and thus are unsuitable for qPCR. We designed primers that amplify a 112 bp fragment of the transcript we described earlier [34] (see Supplementary Figure S2 in the cited reference), which was identical to a transcript (Acc No AY845701.1) that had been revealed in cancer cells by Valgardsdottir et al. (2005) [35]. The amplification product appears as a single band of estimated length both in genomic DNA and polyT cDNA (Figure 7a). In qPCR, its melting curve had a single peak of 79 °C. However, in DNA–DNA FISH on chromosomes, the amplified sequence (HS2/HS3-112) hybridized with more than one chromosome pair (Figure S1). Thus, the designed primers could be used in qPCR because on one hand, the melting curve of the amplification product had a single peak and on the other hand, the amplified sequence was present on more than one chromosome.

In untreated cells, the lowest level of transcription was detected in RPMI8226 cells and the highest level in NR-MSC (Figure 5c). Incubation with TNFα decreased HS2/HS3 transcription in all MSC groups but not in RPMI8226 (Figure 7b). The most drastic downregulation was observed in NR-MSC (fold change: 0.02 ± 0.02 to the untreated samples, *p* < 0.05); in HD-MSC and PoCR MSC, the transcription declined to a lesser extent (HD-MSC: 0.2 ± 0.1 and PoCR: 0.2 ± 0.01 vs. untreated samples). In RPMI8226, HS2/HS3 transcripts quantity increased 2.6 ± 1.1-fold after incubation with TNFα. It is probably that the difference in the initial number of transcripts between MM and MSC cells as well as cells reaction on TNFα reflects a different role played by HS2/HS3 transcripts in MM and MM-MSC cells.

Il-6, another pro-inflammatory cytokine, and VEGF also downregulated HS2/HS3 transcription in MM-MSC. The effect was more prominent in NR-MSC (Figure 7c).

Thus, pro-inflammatory cytokines downregulate HS2/HS3 transcription in MSC, especially in non-responding patients, and upregulate it in MM cells.

## 3. Discussion

TME involves cellular components (e.g., mesenchymal stem/progenitor cells, mature mesenchymal cells, endothelial cells, sympathetic neurons, non-myelinating Schwann cells, perivascular cells, mesodermally derived cells, as well as mature hematopoietic cells such as monocytes, macrophages, regulatory T cells, neutrophils, and megakaryocytes) and soluble factors secreted by these cells [36]. The interaction of MM cells with TME is known to be one of the most important mechanisms for the progression and growth of tumor cells, as well as the development of drug resistance.

In our study, a functional realignment of cellular and extracellular elements involved in the formation of the hematopoietic niche was detected in treated patients’ BM during histological examination regardless of BM tumor infiltration type interstitial or diffuse. We observed an increase in the number of endosteal stromal cells, the density of microvessels in BM parenchyma, including the anatomical location of hematopoietic cells in endost (Figure 1). There was no correlation between genetic aberrations, disease stage, and disease duration. The relationship between the density of microvessels and both clinical and laboratory parameters, marking the adverse course of the disease, was revealed. The vascular network has been recently reconsidered as an important part of the hematopoietic niche and TME [37]. In a normal niche, quiescent hematopoietic stem cells are associated with small arterioles, highly abundant in the endosteum. Activated HSCs migrate away from the proximity to arterioles [38]. The remodeling of the vascular niche in MM has not been studied in detail, however, the crosstalk between cancer and endothelial cells has been demonstrated in other hematological malignancies. It promotes angiogenesis and cancer cells proliferation [37]. In patients with acute myeloid leukemia, BM displays significantly increased microvessels density [39].

The differences were revealed not only in histopathology examination but also in MM-MSC grown ex vivo. The osteogenic differentiation was different in HD and MM patients’ groups (Figure 4). The osteogenic potential declined in all MM-MSC samples. However, the decline was more prominent in samples obtained from NR patients than in those obtained from PoCR patients. Limited ability to osteogenic differentiation is associated with the development of bone lysis in MM which can be caused either by inducing osteoclastogenesis or by inhibiting the differentiation of MSC into osteoblasts from osteogenic precursors [36,40]. Another mechanism for suppressing the differentiation of MSC into osteoblasts is associated with the intensive production of DKK1 by MM cells [41]. In some samples from NR patients, the osteogenic differentiation was similar to HD samples. It can be explained by the survival of the MSC clones without impairing the ability to undergo osteogenic differentiation in cell cultures. MSC are highly heterogenic within a primary culture due to both differences between cells and the methods of obtaining BM samples [42].

MM-MSC and HD-MSC differed also in their proliferation capacity (Figure 3). The observed decline of proliferation rate is consistent with the data of another study on changes of MSC from MM patients. The study revealed a decrease in the proliferation rate, cell enlargement, and other features of senescence of MSC from patients with MM [22,43]. A decrease in the proliferation rate of MM-MSC correlated with the expression of markers of the tumor-associated phenotype (Figure 4) and type of response to treatment.

Decreased proliferation capacity and quality of the osteogenic differentiation reflect changes that occur with MSC after their contact with tumor cells. MSC then acquire features of a tumor-associated phenotype, such as the synthesis of αSMA and SA-β-gal, that are not typical for HD-MSC [43,44].

Tumor-educated MSC survived the treatment and protected MM cells from bortezomib-induced apoptosis, as shown in cell culture experiments (Figure 2). This function can be performed both by HD and MM-MSC. However, MM-MSC, especially NR-MSC, are more effective. The MM patients enrolled in our study had been subjected to treatment regimens that included bortezomib. Unlike MM cells, MSC do not undergo apoptosis after treatment with bortezomib. They survive treatment and, moreover, they can be induced by bortezomib into osteogenic differentiation via Wnt-independent activation of beta-catenin/TCF signaling and/or through activation of the endoplasmic reticulum stress signaling branch Ire1α/Xbp1s [45,46]. In the study performed by Zhang et al. (2020) [46], the viability of BM MSC of healthy mice decreased to almost zero level at 10–15 nm of bortezomib applied either for 24 or 48 h. In our study, the HD-MSC viability dropped down below 60% at 20 nm, and MM-MSC viability remained within the range of 75–90% even at the bortezomib concentration 20 nm. Thus, probably, the MSC sensitivity to the drug is species-specific and/or is increased in patients after treatment with bortezomib. Given that MM-MSC are more effective in protecting MM cells from the bortezomib and more resistant to the drug (Figure 2), their presence in the hematopoietic niche after treatment should be taken into account when developing new therapeutic approaches.

Thus, our experiments give a body of evidence that the hematopoietic niche of NR is different from the niche of patients who responded to treatment. The differences can be observed by histological, immunohistochemical, and other diagnostic methods as well as in vitro experiments on MM-MSC grown in culture. MM-MSC are very similar in their phenotype to CAF (cancer-associated fibroblasts), which can originate from multiple cell lineages such as resident fibroblasts, MSC, endothelial cells, and hematopoietic stem and progenitor cells via the endothelial–mesenchymal transition [47]. CAF interact with MM cells through cell contacts and their secretome. CAF from bortezomib-resistant patients are resistant in vitro to the drug and prevent the bortezomib-induced apoptosis of co-cultured MM cells [48].

Repetitive non-coding elements of the human genome are now considered functionally important sequences involved in many cellular processes [14]. Both tandemly repeated and dispersed repeats DNA and RNA are involved in solid tumor progression [27,30,31,49]. We analyzed MM transcriptomes and revealed remarkable changes in the TR transcription profile (Figure 5a,b). Both centromeric and pericentromeric repeats are transcriptionally activated but the maximal increase was observed in samples of NR patients with progressive disease and in samples of MM patients that contained cells of mesenchymal non-hematopoietic lineage. In silico, HS2/HS3 satellites were remarkably upregulated. In qPCR and immunohistochemical studies, the HS2/HS3 transcripts were detected exclusively in MM-MSC and the signal was more prominent for MM-MSC of NR. We demonstrated previously that transcription and decondensation of HS2/HS3 was a hallmark of senescent cells [18]. Senescence increases the tumor-supporting properties of MSC [1,26]. Transcription of pericentromeric DNA (HS2/HS3) was demonstrated for different types of solid epithelial tumors [16,17] but has not been shown for MM, although epigenetic reprogramming was observed by Bollati et al. [20]. In our study, pericentromeric satellites were transcribed in MM-MSC, but not in MM cells. Moreover, co-culturing of MM cells and HD-MSC induced the transcription of HS2/HS3 in MSC. Thus, contact of normal HD-MSC with tumor cells might lead to changes in the transcriptional activity of the non-coding part of the genome. One major mechanism through which non-coding RNAs affect tumorigenesis is by binding to proteins required for maintaining genomic stability, such as chromatin modifiers and DNA damage response factors [17,50,51]. It was demonstrated that a key subset of these lncRNAs functioned as immunostimulatory “self-agonists” and directly activated cells of the mononuclear phagocytic system to produce pro-inflammatory cytokines [52]. Solovyov et al., found that global repeat derepression, including the human satellite repeats, correlates with an immunosuppressive phenotype in colorectal and pancreatic tumors [31]. Transcription of pericentromeric heterochromatin was also observed by Evdokimova et al., in solid tumor fibroblasts which correlated with the metastatic progression [30]. These data suggest another possible mechanism of satellite transcripts’ involvement in cancer progression. In late-stage tumors, in which abundant repetitive element expression is associated with failure of tumor suppressors, the large-scale transcription of many ‘non-self’ repetitive elements was co-opted by the tumor’s evolution to maintain an advantageous inflammatory state. The distinct sequence motifs in satellite RNAs, including human satellites, that appear ‘non-self’, led to differential innate immune responses [31,52]. In MM-MSC cell cultures in vitro we could observe cytoplasmic or extracellular HS2/HS3 transcripts very rarely (e.g., Figure 6). However, extranuclear localization of HS2/HS3 transcripts was demonstrated by Bronkhorst et al. for osteosarcoma cells [53] and in our previous work on lung adenocarcinoma cancer-associated fibroblasts [28].

Treatment with TNFα decreased the level of HS2/HS3 transcription in MM-MSC and increased in MM cells (Figure 7b,c). IL-6 and VEGF also downregulated HS2/HS3 transcription in HD and MM-MSC. The most striking downregulation was observed in NR-MSC. Potent pro-inflammatory cytokines, IL-6 and TNFα, are considered survival factors for MM being capable of decreasing MM cells apoptosis. IL-6 also increases transcription of angiogenic factors, the JAK/STAT pathway, and activates the RAS/MAPK cascade. TNFα increases transcription of prosurvival factors, plasma cells proliferation [33], and adhesion of MM cells to BM MSC along with upregulation of IL-6 in MSC [54]. In MSC, TNFα promotes heterochromatin silencing of the promoter of Runt-related transcription factor (RUNX)2 and thus impairing osteogenesis [55]. It has recently been shown that TNFα, transcribed by CD8+ stem cell memory T (Tscm) cells and interferon-responsive effector T cells, induces a transition from MSC to inflammatory MSC (iMSC) in the BM of patients after the first-line treatment. iMSC are involved in the regulation of cycling plasma cells and the modulation of myeloid cell function. The authors showed that bone marrow inflammation is not reverted by successful antitumor therapy, suggesting a role for iMSC and bone marrow inflammation in disease persistence or relapse [11]. Hence, in MM cells and MSC, the same cytokines play different roles and produce different biological effects. Furthermore, the different effects of cytokine TNFα might be caused by the different initial quantity of transcripts in MM and MSC cells: the strongest downregulation was observed in NR-MSC where the maximal level of transcription was registered before the treatment with TNF-a. This result might reflect the existence of a regulatory mechanism aimed at a balanced TR DNA transcription in cells.

The obtained data confirmed that HS2/HS3 transcription is associated with the senescent TME phenotype and is possibly involved in the mechanisms of drug resistance in hematological malignancies.

## 4. Materials and Methods

### 4.1. Ethics

All BM samples were obtained from healthy donors and patients with MM in line with the WMA Helsinki Declaration (Declaration of Helsinki: Ethical Principles for Medical Research Involving Human Subjects, including amendments made by the 64th Meeting of WMA in Fortaleza, Brazil, October 2013) (https://www.wma.net/policies-post/wma-declaration-of-helsinki-ethical-principles-for-medical-research-involving-human-subjects/ (last accessed on 2 February 2022)) [56]. The study was approved by the ethical committee of the Russian Scientific Institute of Hematology and Transfusiology (protocol No 6-2019, 11 June 2019). The written informed consent was obtained from each patient participating in the study.

### 4.2. Patients

Twelve patients after bortezomib-based induction therapy (the first phase of treatment for multiple myeloma) performed according to the Treatment Standards of the Russian Federation, (regimens: bortezomib–cyclophosphamide (CV), bortezomib–cyclophosphamide–dexamethasone (VCD), bortezomib–lenalidomide–dexamethasone (VRD) followed by autologous hematopoietic stem cell transplantation), were recruited in the study. The detailed information about the patients enrolled in the study is given in Table 1.

Patients’ age ranged from 49 to 71 years with a median of 61 years. The MM response criteria were based on the “National clinical recommendations on diagnosis and treatment of multiple myeloma” [57]. In the revised criteria, the percentage of BM PC is one of the key parameters for the differentiation of monoclonal gammopathy of undetermined significance (MGUS) and MM. The most important criterion in MM diagnosing is the clonal PC percentage in the BM > 10% [58,59]. Therefore, all the patients were divided into 2 groups: with partial or complete response (PoCR) to treatment (less than 10% of PC in an aspirate, a group of 9 patients) and NR, i.e., after unsuccessful treatment (from 10% to 82.4% of PC in an aspirate, a group of 3 patients).

### 4.3. Histopathological Examination

Trephine biopsies of the BM from the patients with confirmed MM were fixed in buffered 10% neutral buffered formalin, embedded in paraffin, cut into 3 μm sections. Deparaffinized and rehydrated sections were stained with hematoxylin and eosin (H&E) using standard techniques.

Immunohistochemical studies (IHC) were carried out to identify tumor cells and morphological features of the BM microenvironment. After antigen retrieval sections were incubated with antibodies against CD138 (RTU, Agilent, Dako, Carpinteria, CA, USA) to reveal the PC, CD34 cl. II (RTU, Agilent, Dako, Carpinteria, CA, USA) to reveal the microvessels, CXCR4 (1:200, ab124824, Cambridge, UK), α-SMA (1:400, ab7817, Abcam, Waltham, MA, USA) for 30 min at room temperature followed by EnVision FLEX system (high pH (Link), HRP, Rabbit/Mouse, Agilent, Dako, Carpinteria, CA, USA) for 30 min at room temperature. A 3,3-diaminobenzidine (Agilent, Dako, Carpinteria, CA, USA) was used as the chromogen. Finally, the slides were counterstained with hematoxylin and mounted after dehydration.

Morphometric analyses. Each parameter was scored quantitatively by a blinded observer who examined 10 fields of view (200× *g* magnification) in one section for each slide using VideoTesT-Master (Morphology) software (VideoTesT, Saint Petersburg, Russia).

### 4.4. Bioinformatics

Differential analysis of TR DNA transcription in MM patients was performed using single-cell transcriptomes published by Ruy et al. (2020) [29] (https://www.ncbi.nlm.nih.gov/bioproject/PRJNA415945, last accessed on 5 January 2022) and by de Jong et al. (2021) [11] (https://www.ebi.ac.uk/arrayexpress/experiments/E-MTAB-9139/, last accessed on 10 January 2022). Transcripts per million (TPM) value was calculated for all TR sequences using Kallisto software v0.46.1 (Berkeley, CA, USA) [60]. The full set of TR sequences was taken from Repbase ver. 23.12 [61]. Heatmaps were plotted using pheatmap for R software (https://github.com/raivokolde/pheatmap (accessed on 5 January 2022) and GraphPad Prism v7.0 software (San Diego, CA, USA). 

### 4.5. Cell Cultures

BM samples (1–6 mL) from MM patients (*n* = 12) were obtained by sternal puncture during secondary treatment diagnostics. MSC from HD (HD-MSC) were obtained from iliac crest punctures of 3 HD (41–50 years).

All the BM samples were diluted with saline in a ratio of 1:3 (*v/v*, including the anticoagulant volume). Mononuclear cells were isolated following the standard Ficoll-Paque (ρ = 1.077 g/cm^3^, PanEco, Moscow, Russia) density gradient protocol. Briefly, the diluted BM sample (7.5 mL) was layered on 7.5 mL of Ficoll-Paque and centrifuged in 15 mL tubes for 40 min at 400 g without brake. The mononuclear cell fraction (buffy coat) was collected, diluted with PBS (1:10), and centrifuged at 200 g for 10 min to remove Ficoll and platelets. Cells were then plated in 25 cm^2^ flasks and expanded in DMEM with low glucose (Thermofisher, Waltham, MA, USA) supplemented with 10% Advanced Stem Cell Supplement (HyClone, Logan, UT, USA) as well as 100 units/mL penicillin and 100 μg/mL streptomycin (Gibco, Waltham, MA, USA) at 37 °C in a 5% CO_2_ and 7% O_2_ atmosphere, because, for most tissues, the physiological O_2_ concentration does not exceed 8%, for bone 6.6–8.6 [62,63,64]. MSC adhered in 5–7 days, and nonadherent cells were discarded during the first passaging. The medium was changed every 3 days. When reaching 70–80% confluency, cells were harvested using trypsin/Versen solutions (Gibco, Waltham, MA, USA) and split at a 1:2 ratio.

Human multiple myeloma RPMI 8226 cells [65] were cultured in DMEM medium supplemented with 10% fetal bovine serum (HyClone, Logan, UT, USA) as well as 100 units/mL penicillin and 100 μg/mL streptomycin (Gibco, Waltham, MA, USA) at 37 °C in the same hypoxic atmosphere as described above. Primary cell culture of human foreskin fibroblasts of a boy of 4 y.o. was kindly given by Dr. N.M. Yudintceva (Institute of Cytology, Russia) and was cultured in DMEM supplemented with 10% fetal bovine serum (HyClone, Logan, UT, USA) as well as 100 units/mL penicillin and 100 μg/mL streptomycin (Gibco, Waltham, MA, USA) at 37 °C in a 5% CO_2_ atmosphere. The medium was changed every 3 days. Cytokines IL-6 (Sci-Store, Moscow, Russia), VEGF (Sci-Store, Moscow, Russia), TNF-a (Stem Cell Technologies, Cambridge, MA, USA) were added to a final concentration of 100 ng/Ml and incubated for 48 h. Then the cells were harvested and proceeded to RNA isolation procedure and used for qPCR.

### 4.6. Co-Culturing with RPMI 8226

The interaction of MSC and MM cells in the hematological niche was mimicked in co-culture experiments. MSC obtained from HD patients were co-cultured with RPMI 8226 cells under non-contact and contact conditions. MSC were seeded at a density of 10^5^ cells per well in 6-well plates. In 24 h, RPMI 8226 cells were added at a ratio of 1:10 in 3 wells while 3 other wells were used as a control. In non-contact experiments, RPMI 8226 cells were seeded into cell culture transwell inserts with a pore diameter of 0.4 µm (Sarstedt, Nümbrecht, Germany), which permitted transport of organic and inorganic molecules only. In contact co-cultures, RPMI 8226 were seeded without inserts. On the 3rd day of co-culturing, the MSC were fixed as described below for immunoFISH experiments.

Bortezomib (5–20 nm), a proteasome inhibitor, one of the anticancer drugs included in MM standard treatment regimes, was added to cell cultures. Cells from upper (RPMI 8226) and lower (MSC) wells were harvested in 48 h, stained with annexin-FITC and propidium iodide (both from Sigma-Merck, Darmstadt, Germany) according to the manufacturer’s protocol, and subjected to flow cytometry. Cells negative in annexin and propidium iodide staining were considered live cells.

### 4.7. RNA Isolation and Quantitative PCR

Total RNA from MSC or RPMI8226 was isolated using GenElute Mammalian Total RNA Miniprep Kit (Sigma, Burlington, MA, USA). Total RNA (1 µg) was reverse-transcribed with MMLV RT kit (Evrogen, Moscow, Russia). Real-time PCR was performed with 50 ng cDNA and SYBRGreen PCR Mastermix (Evrogen, Moscow, Russia) using CFX96 Real-Time System (Bio-Rad, Hercules, CA, USA). The thermocycling conditions were as follows: 95 °C for 5 min, followed by 40 cycles at 95 °C for 10 s, 58 °C for 20 s and 72 °C for 30 s (a 3-steps protocol is recommended by the PCR Mastermix manufacturer). A final heating step of 65 °C to 95 °C was performed to obtain melting curves of the final PCR products. mRNA expression levels were calculated by the 2^−ΔΔCt^ method with the levels of gene transcription normalized to the housekeeping genes *GAPDH* encoding glyceraldehyde 3-phosphate dehydrogenase (GAPDH). Primers for HS2/HS3 amplification were targeted to the most abundant HS2/HS3 transcript we described earlier in oocyte transcriptomes [34], in senescent fibroblasts and some cancer lines [18], and which was also detected by [35]. The following HS2/HS3 primers were designed to amplify a 112 bp fragment at the 3′end of the transcript: ZhF, 5′-CGT TTC CTT TCG ATG GCG TT-3′ and ZhR, 5′-TGA AAT CCA ATA TGA TCA TCA TCG AA-3′. The following primers were used to amplify the *GAPDH* reference gene: forward, 5′-AGGTCGGAGTCAACGGATTT-3′, and reverse 5′-TTCCCGTTCTCAGCCTTGAC-3′.

### 4.8. Proliferation Rate Assay

MSC (passage 2–3) were seeded at a density of 10^5^ cells per well in six-well plates. On days 1, 3, and 5, the cells were detached with trypsin and their number was counted in a cell counting chamber (Minimed, Bryansk Russia) and Luna Cell counter (Logos Biosystems, Gyeonggi-do, Korea).

### 4.9. Osteogenic Differentiation Assay

MSC at the 3rd passage were seeded at a density of 10^5^ per well into six-well plates as described above. When the cells reached 90–100% confluency, the medium was changed to MSCgo™ Osteogenic (BioInd, Beit Haemek, Israel) for 21 days in order to induce osteogenic differentiation. Cells were then fixed with 10% PFA for 30 min at room temperature and calcifications were detected by Alizarin Red (Sigma-Aldrich, Burlington, MA, USA) staining for 45 min according to the standard protocol.

### 4.10. Galactosidase Staining

MSC at 5th passage from MM patients and HD were assayed for senescence-associated β-galactosidase (SA-β-gal) as described previously [66]. Human foreskin fibroblasts in late passages treated with doxorubicin (0.1 μg/mL) were used as a positive control [67]. Cells were fixed with 4% PFA for 5 min at room temperature and then incubated with SA-β-gal staining solution overnight in the dark at 37 °C. The reaction was stopped by removing the solution and washing the cells with distilled water. The cells positive for SA-β-gal were of dark blue color.

### 4.11. Immunofluorescence Staining

MSC were grown on cell culture coverslips (SPL Life Sciences, Gyeonggi-do, Korea). Cells were then fixed with 4% PFA for 40 min, permeabilized with 0.1% (*v*/*v*) Triton X-100 for 10 min. The sites of non-specific binding were blocked with a solution containing 3% bovine serum albumin and 0.5% Tween-20 for 30 min. Incubation with FITC-conjugated anti-α-smooth muscle actin (αSMA) antibody (1:200, clone 1A4, Sigma-Aldrich, Burlington, MA, USA) was held for 1 h at 37 °C in a wet chamber. Cells were then washed and mounted with Slowfade^®^ antifade medium containing DAPI (Thermofisher, Waltham, MA, USA).

### 4.12. Flow Cytometry

MSC were immunostained with the following panels of monoclonal antibodies against CD44-FITC/CD73-PE/CD90-PC5/CD105-PC7 and CD34-FITC/CD117-PE/CD14-PC5/CD45-PC7 (Beckman Coulter, Brea, CA, USA) at the 2nd passage. Analysis was performed using a Navios flow cytometer, equipped with 2 diode lasers (488, 638 nm) and 8 detectors, according to standard protocols recommended by the manufacturer (Beckman Coulter, Brea, CA, USA).

RPMI 8226 cells were tested for PC markers to prove that the cell line can be used as MM cells in co-culture experiments. The measurement was carried out on a Navios flow cytometer (Beckman Coulter) equipped with 3 lasers (405, 488, and 638 nm), 10 fluorescence detectors, and the original standard set light filters (Blue Laser: 525/40, 575/30, 620/30, 695/30, 755LP; Red Laser: 660/20, 725/20, 755 LP; Violet Laser: 450/50, 550/40). For PC markers evaluation, staining with a commercial DuraClone RE PC Tube antibody mixture (Beckman Coulter, B80394) was performed, which was a ready-to-use tube with CD38 Pacific Blue, CD45 Krome Orange, CD81 FITC, CD27 PE, CD19 PE-Cy5.5, CD200 PE-Cy7, CD138 APC, and CD56 APC-Alexa Fluor 750 dry antibodies placed on the bottom of the tube. The cell suspension (100 μL) was placed in a DuraClone RE PC Tube, incubated for 15 min at room temperature (20–23 °C), washed by adding 3 mL of DPBS with further centrifugation at 300 g for 7 min, resuspended in 500 μL DPBS, and immediately analyzed on a flow cytometer.

### 4.13. DNA-FISH, DNA–RNA FISH, and immunoFISH

To investigate the presence of HS2/HS3 transcripts DNA–RNA FISH was carried out. An oligonucleotide DYZ1 (5′-tccattccattccattccattccattccattccattccattccattcc-3′) was labeled with the Cy3 fluorochrome (Evrogen, Moscow, Russia). The probe was described previously by Lee et al. [68] (line 1637, Supplementary Table S6 of the cited reference) as the one located in the pericentromeric region of the Y chromosome, but this sequence can also be found in the HS2 and HS3 regions of other chromosomes according to BLASTA. The DYZ1 probe shares 100% homology with several regions in the chromosome 9 sequence PTRS (Acc No DQ980028.1) that we had sequenced earlier [18].

The TR DNA families are chromosome-specific. However, in this study, we were interested in a probe that could hybridize TR transcripts from different chromosomes to reveal different transcripts. According to the in silico data, the DYZ1 probe seemed to be a good candidate. Nevertheless, we verified the in silico data by experiments in situ. Before starting DNA–RNA FISH experiments, the chromosome specificity of the probe was studied on MSC metaphase spreads with DNA-FISH. Before hybridization slides were washed in 2× sodium saline citrate (SSC), denatured in 70% formamide in 2 × SSC at 74 °C for 4 min, and dehydrated by passing the specimens through a series of ethanol solutions. Hybridization was performed at 41 °C with a hybridization mix containing 1 μg/mL of labeled HS2/HS3, 2 × SSC, 30% formamide, 10% dextran sulfate, and 1 μg/mL salmon sperm DNA. Slides were then washed with 2 × SSC at 41 °C and 1 × SSC at room temperature for 10 min each, with 0.5 × SSC and 0.25 × SSC both at room temperature for 5 min. The slides were then rinsed in distilled water and mounted with Slowfade^®^ mounting medium containing DAPI (Invitrogen, Waltham, MA, USA).

For DNA–RNA FISH cells were fixed with 2% PFA at room temperature for 30 min, washed with 2 × SSC, and dehydrated. The denaturation step was omitted. The hybridization and post-hybridization washings were performed as described above.

For DNA–RNA FISH on paraffin-embedded sections, they were deparaffinized in xylene and rehydrated in ethanol with increasing water concentration and then dehydrated again in ethanol to proceed slides for FISH.

In immunoFISH experiments, the DNA–RNA FISH step was performed as described above. After the washing in 0.25 × SSC, the cells were subjected to staining with the AB against αSMA as described in 4.9.

### 4.14. Microscopy

Image acquisition was performed using an Olympus FV3000 confocal microscope (Olympus, Tokyo, Japan). To detect DAPI, FITC, and Cy3, the 405, 488, and 561 nm diode lasers were used for excitation, respectively. The cells were sectioned in the *z*-axis with a 0.7 μM interval. Image acquisition for proliferation assay, SA-β-gal staining, and calcifications estimation was performed with an Axio Vert.A1 microscope (Carl Zeiss, Rossedorf, Germany). The histological slides were observed and photos were taken using an optical microscope (Nicon Corp., Tokyo, Japan) at 100× magnification. For each slide, images of at least five random visual fields were taken.

Quantification of images was performed using Fiji software [69]. Before processing, images were calibrated the ratio physical dimensions/number of pixels was calculated. Images of a single channel were processed with Subtract and Unsharp mask instruments of the software and were further converted to grayscale. Using the Threshold tool, a threshold value of gray was set and the image was converted to black-and-white the values below the threshold were converted to black, above it to white pixels. The nuclei were manually selected using the Freehand selection tool. The number of white pixels per nucleus was calculated. A similar approach was used in Corel PHOTOPAINT software vX7 (Ottawa, ON, Canada) the cell areas were manually selected and the number of pixels above a threshold in the channel of interest was obtained from the image histogram. In this case, the step of conversion to black-and-white was omitted to eliminate possible signal loss during conversion. The results of both approaches were compared using a one-way ANOVA test. When no significant difference was observed, the Fiji results were used for chart plotting and statistical calculations.

### 4.15. Statistical Analysis

The data are representative of three or more independent experiments. qPCR was performed in three technical and three biological replicates. GraphPad Prism v 7.0 software (San Diego, CA, USA) was used. Results are reported as the mean ± SD. Comparisons between groups were made using t-test or Kruskal–Wallis test for histological studies, one-way ANOVA was used in fluorescence images and qPCR studies. Pearson correlation coefficient (r) was measured in histopathological examination. Significant difference was assessed with a *p*-value < 0.05.

## 5. Conclusions

The phenotype of successfully treated MM patients’ MSC differed from one of the HD being closer to the CAF phenotype. The MSC of NR patients had a prominent cancer-associated phenotype. The upregulation of pericentromeric DNA transcription in cancer-associated MM-MSC of NR observed in our study was not demonstrated previously. Pericentromeric non-coding DNA transcription is associated with niche impairment in patients with ineffective or partially effective multiple myeloma treatment.

## Figures and Tables

**Table 1:** Information about the patients enrolled in the study.

PC < 10% (*n* = 9)	**Type of Response**	**Diagnosis**	**Type of M Protein**	**BM Infiltration**	**Microvessel Density of BM**	**Myelogram, Percentage of PC**	**M-Component in Serum (in Urine for Bence-Jones Protein), g/L**	**Karyotype**	**MRD**	**Treatment**
	SD	non-secretory MM	−	10%	7.20%	6%	0	No abnormalities	+	4 VCD courses
	CR	IIA	Ig G κ pa	10%	8.90%	3%	0	No abnormalities	+	6 VCD courses, aHSCT
	PR	IIIA	Ig A κ	5%	10.20%	2.40%	4	No abnormalities	+	5 VCD courses
	CR	IIIB	Ig G λ	1–2%	7.40%	1%	0	No abnormalities	+	5 VCD courses, aHSCT
	VGPR	IIIA	Ig G κ	3%	8.50%	3.60%	10.7	No abnormalities	-	2 VRD courses + 2 KRd courses, aHSCT
	VGPR	IIIA	Ig G κ	30%	11.70%	2.20%	7.7	13q14 and 13q34 deletions	+	3 VCD courses, aHSCT
	PR	IIIA	Ig G κ	1–2%	10.60%	1.40%	15	No abnormalities	+	5 VCD courses, aHSCT
	CR	Bence-Jones MM IIIA	Ig G κ	1–2%	8.60%	2.60%	0	No abnormalities	-	6 VD courses
	VGPR	IIA	Ig G λ	1–2%	9.20%	2.80%	0	No abnormalities	-	VRD, aHSCT
Non-responders (*n* = 3)	SD	IIIA	Ig G κ	50%	13.00%	47%	0.47	14q32 monosomy, 13q14 and 13q34 deletions, TP53/17p13 deletions or monosomy	+	6 VD course, aHSCT
	SD	IIIA	Ig A κ	50%	12.50%	14.40%	6	No abnormalities	+	3 VCD course, aHSCT
	SD	IIIA	Ig G λ	90%	11.40%	82.40%	0	Y chromosome loss	+	2 VCD course

Abbreviations: CR = complete response; aHSCT = autologous hematopoietic stem cell transplantation; PR = partial response; SD = stable disease; VGPR = very good partial response. Regimens abbreviations: CV= bortezomib–cyclophosphamide; VCD = bortezomib–cyclophosphamide–dexamethasone; VD = bortezomib–dexamethasone; VRD = bortezomib–lenalidomide–dexamethasone; KRd = karfilzomib–lenalidomide–dexamethasone.

## Data Availability

Not applicable.

## Supplementary Materials

The following supporting information can be downloaded at: https://www.mdpi.com/article/10.3390/ijms23063359/s1.
