# Supplementary material for: Pericentromeric Non-Coding DNA Transcription Is Associated with Niche Impairment in Patients with Ineffective or Partially Effective Multiple Myeloma Treatment
Source: Int J Mol Sci. 2022 Mar 20;23(6):3359. doi: 10.3390/ijms23063359 (PMC8951104; doi:10.3390/ijms23063359)
Supplement: Supplementary file 1 [file ijms-23-03359-s001.zip › ijms-1626581-supplementary.pdf]

International Journal of *Molecular Sciences*

Article

Pericentromeric Non-Coding DNA Transcription Is Associated with Niche Impairment in Patients with Ineffective or Partially Effective Multiple Myeloma Treatment

Natella I. Enukashvily [1,2,*], Natalia Semenova [3,*], Anna V. Chubar [1], Dmitry I. Ostromyshenskii [1], Ekaterina A. Gushcha [1], Sergei Gritsaev [3], Stanislav S. Bessmeltsev [3], Viktor I. Rugal [3], Egor M. Prikhodko [4,5], Ivan Kostroma [3], Anastasia Zherniakova [3,†], Anastasia V. Kotova [1], Liubov A. Belik [1], Alexander Shumeev [4,‡], Irina I. Maslennikova [2,4] and Dmitry I. Ivolgin [2,4]

[1] Lab of the Non-coding DNA Studies, Institute of Cytology, Russian Academy of Sciences, 194064 St. Petersburg, Russia; annachubar95@incras.ru (A.V.C.); necroforus@gmail.com (D.I.O.); gushcha.ekaterina@gmail.com (E.A.G.); anastkotova@gmail.com (A.V.K.); l.a.belik@list.ru (L.A.B.)
[2] Cell Technologies Lab, North-Western State Medical University Named after I.I. Mechnikov, 191015 St. Petersburg, Russia; Irina.Maslennikova@szgmu.ru (I.I.M.); Dmitrii.Ivolgin@szgmu.ru (D.I.I.)
[3] Clinical Department, Russian Research Institute of Hematology and Transfusiology FMBA of Russia, 191024 St. Petersburg, Russia; gritsaevsv@mail.ru (S.G.); bessmeltsev@yandex.ru (S.S.B.); sciencerugal@gmail.com (V.I.R.); obex@rambler.ru (I.K.); nasta2045@yandex.ru (A.Z.)
[4] Pokrovsky Stem Cell Bank, LLC, 199106 St. Petersburg, Russia; ceo@pokrovcell.ru (E.M.P.); a-shu@yandex.ru (A.S.)
[5] Faculty of Clinical Propaedeutics, North-Western State Medical University Named after I.I. Mechnikov, 191015 St. Petersburg, Russia
* Correspondence: n.enukashvily@incras.ru (N.I.E.); semenova@mlc-lab.ru (N.S.)
† Present affiliation: Clinica Universidad de Navarra, Centro de Investigacion Medica Aplicada (CIMA), Instituto de Investigacion Sanitaria de Navarra (IDISNA), 31008 Pamplona, Spain
‡ Present affiliation: Core Facility for Cell Technologies and Immunology, 'Sirius' University, 354340 Sochi, Russia

Citation: Enukashvily, N.; Semenova, N.; Chubar, A.; Ostromyshenskii, D.; Gushcha, E.; Gritsaev, S.; Bessmeltsev, S.; Rugal, V.; Prikhodko, E.; Kostroma, I.; et al. Pericentromeric Non-Coding DNA Transcription Is Associated with Niche Impairment in Patients with Ineffective or Partially Effective Multiple Myeloma Treatment. *Int. J. Mol. Sci.* **2022**, *23*, 3359. https://doi.org/10.3390/ijms23063359

Academic Editor: Arianna Scuteri

Received: 21 February 2022
Accepted: 17 March 2022
Published: 20 March 2022

Publisher's Note: MDPI stays neutral with regard to jurisdictional claims in published maps and institutional affiliations.

Copyright: © 2022 by the authors. Licensee MDPI, Basel, Switzerland. This article is an open access article distributed under the terms and conditions of the Creative Commons Attribution (CC BY) license (https://creativecommons.org/licenses/by/4.0/).

Supplementary Material

Supplementary Table S1. The number of cells expressing positive and negative markers of MSC in cultures obtained from HD and MM patients with different PC percentage.

Surface antigens		MM-MSC from PoCR patients	MM-MSC from NR patients	HD-MSC
Markers of MSC	CD44	97,9 ± 4,9	99,4 ± 0,9	97,5 ± 2,3
	CD73	98,7 ± 3,2	99,2 ± 1,3	95 ± 3,2
	CD90	98,4 ± 3,3	97,5 ± 2,8	99 ± 0,4
	CD105	98,1 ± 4,5	99,2 ± 1,4	93,3 ± 6,5
Markers of hematological cells	CD14	3,3 ± 3,2	0,7 ± 0,6	1,5 ± 0,8
	CD34	0,3 ± 0,8	0,6 ± 1,1	3,1 ± 2,8
	CD45	2,3 ± 2,5	0 ± 0	1,9 ± 1,6
	CD117	2,7 ± 3,4	1,1 ± 1,1	1,1 ± 0,4

Abbreviations: HD = healthy donors; HD-MSC = mesenchymal stromal cells from healthy donors; MSC = mesenchymal stromal cells; MM = multiple myeloma; MM-MSC = mesenchymal stromal cells from multiple myeloma patients; NR = non-responder; PC = plasma cells; PoCR = partial or complete response.

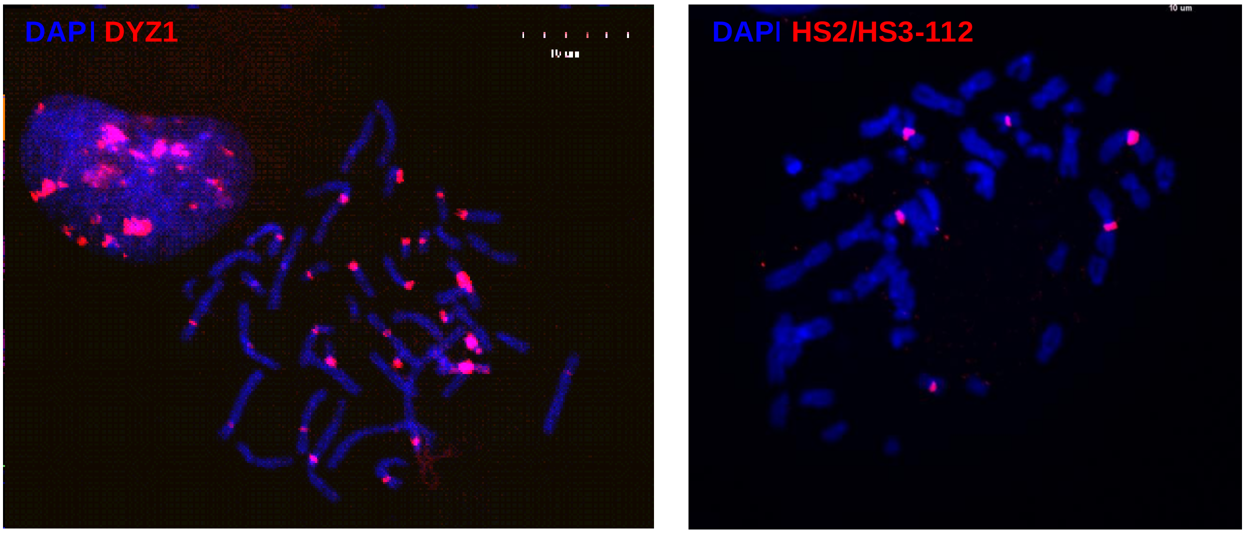

Supplementary Figure S1. Chromosome specificity of two HS2/HS3 probes (red) used in the study. DNA-DNA FISH was performed on metaphase plates as described in Materials and Methods.
